# Aid Policy and Australian Public Opinion

**DOI:** 10.1002/app5.230

**Published:** 2018-04-16

**Authors:** Terence Wood

**Affiliations:** ^1^ Development Policy Centre, Crawford School of Public Policy The Australian National University Acton Australian Capital Territory Australia

**Keywords:** aid, public opinion, Australia, aid policy

## Abstract

Since 2013, Australian aid has been reduced and increasingly focused on delivering benefits to Australia. Motivated by these changes, this paper fills three gaps in the existing literature on public opinion about aid. It provides the only recent detailed study of Australians' opinions about aid. It studies specific policy questions in addition to the broader questions typical of international research. And it studies views on the purpose of aid, an area not previously researched. Although Australians are generally supportive of aid, most backed major aid cuts in 2015. However, most Australians think the purpose of Australian aid should be helping people in poor countries, not bringing benefits to Australia. There is a clear left–right divide in responses to all questions; however, some variables correlated with support for aid fail to explain variation in views about aid's purpose. The paper concludes by discussing ramifications for those who seek to change aid policy.

## 1 Introduction

The election of the centre‐right Coalition government in 2013 brought major changes to Australian aid. The government aid budget was repeatedly cut culminating with the largest ever cuts to Australian aid at the beginning of the 2015–16 financial year (Howes & Pryke 2014). The Coalition government has also placed an increased emphasis on giving aid to advance Australia's own interests rather than helping developing countries (Wood et al. 2017, p. 244).

Although public opinion is not the only factor that shapes high‐level aid policy decisions, there is international evidence that suggests that the views of the public play a contributing role in the choices politicians make about aid policy (Chong & Gradstein 2008; Heinrich et al. 2016; Milner & Tingley 2010; Milner & Tingley 2011; Prather 2011; Stern 1998). In Australia, politicians have repeatedly emphasised the need for public support if the aid budget is to be grown again (for example, Bishop 2017; Fierravanti‐Wells 2017).

Policymakers, academics and those seeking to influence aid policy in Australia currently have two different types of information that they can draw upon as they seek to better understand Australians' views on aid. The first of these is Australian polling conducted by organisations such as the Lowy Institute (e.g. Oliver 2017, p. 16). The second is international academic work on aid and public opinion (e.g. Chong & Gradstein 2008; Diven & Constantelos 2009; Henson & Lindstrom 2013; Paxton & Knack 2012). 1Two Australian studies have conducted more sophisticated analyses of survey data about opinions on aid; however, one study (Kelley 1989) is nearly 20 years old and the unit of analysis in the other paper (Wood et al. 2016) is electoral districts, not individual Australians. However, both of these different types of information have limitations for someone seeking a comprehensive understanding of public views about aid policy in Australia. A key limitation of polls such as those run by the Lowy Institute is that they provide only basic aggregate information on levels of support. Although this is sometimes accompanied by simple cross tabulations looking at bivariate relationships between particular respondent traits and views about aid, no serious attempt is undertaken to control for confounding variables when identifying traits associated with different views about aid. As a result, because of the risk of spurious relationships, these types of polls reveal little about the traits most likely to drive different views about aid in Australia.

Many international studies, on the other hand, do carefully control for the influence of different variables and do identify important traits associated with different views about aid. However, these studies have their own particular limitation for the Australian audience: none focus on Australia. This is an issue both because support for aid varies considerably between countries, and because the relationships between different traits and views about aid also vary (Clarke et al. 2014; Paxton & Knack 2012). Much of the international work also draws on data from very general questions, at least one degree removed from the actual policy decisions public opinion is thought to shape (Hudson & vanHeerde‐Hudson 2012). Typically, survey participants are not told how much aid their country gives nor are policy options and trade‐offs made clear as survey participants' views are sought (Hudson & vanHeerde‐Hudson 2012). Instead, the staple of the international literature are questions such as the following (used by Chong & Gradstein 2008; Clarke et al. 2014; Diven & Constantelos 2009; Paxton & Knack 2012):

Some people favour, and others are against, having this country provide economic aid to poorer countries. Do you think that this country should provide more or less economic aid to poorer countries?

Another limitation both of polls such as the Lowy Poll and international scholarly work is that, almost without exception, these undertakings focus either on support for aid or views about aid volume and do not analyse data from questions that focus on people's preferences about the purpose of aid giving. However, in Australia, the increasing focus on using Australian aid to bring benefits to Australia has become a major policy issue (Wood et al. 2017, p. 244). Australia is not alone in this; international work has shown mixed motives with respect to the purpose of aid to be a fraught area for many donors (Alesina & Dollar 2000; Heinrich 2013; Hoeffler & Outram 2011).

In this paper, I draw on data from two public opinion surveys to provide a detailed picture of the Australian public's views on aid. As I do this, I address the issues that I have just outlined. I use Australian data. I study broad support for aid, and I study responses to a more specific policy‐relevant question about the 2015 aid cuts. I also study responses to a question about the purpose of aid. In addition to providing findings based on aggregate responses to these questions, I use regression analysis to identify the socio‐demographic traits and beliefs associated with support for aid, opposition to aid cuts, and preferred purpose of aid giving.

Looking at aggregate results, I find strong public support for Australia giving aid, but I also find that this support did not translate into opposition to the 2015 aid cuts—most Australians favoured the cuts over policy alternatives. I also find that a substantial majority of Australians want aid given for the purpose of helping developing countries rather than for the sake of advancing Australia's geostrategic and commercial interests. My regression results are similar, but not identical, when comparing general support for aid and opposition to aid cuts. Most clearly, academic education and left‐leaning political views are associated with both support for aid and opposition to cuts, while age is associated with opposition to aid and support for cuts. There are clear differences, however, in some of the traits associated with support for aid and those associated with the belief that the purpose of Australian aid giving should be humanitarian, and not about bringing benefits to Australia. For example, while age is negatively associated with support for aid, it is positively associated with the preference that aid be given for humanitarian ends, and not focused on bringing benefits to Australia.

The rest of this paper is structured in the following way. First, I cover existing international work on public opinion about aid. Although this work is often limited by the questions it draws upon, it still points to a useful set of variables for inclusion in regression analysis. Following this review, I detail my methods. I then move onto findings, first looking at aggregate level responses before moving to regression analysis. In the conclusion, I discuss the ramifications of what I have found for those who seek to change high‐level aid policy in Australia. (Replication files can be downloaded from https://goo.gl/ufGzC2.)

## 2 Existing Research on Public Opinion and Aid

An emerging international literature has identified a range of traits associated with people's opinions about aid. In this section, I cover the main findings of existing work. This is done to identify variables that might be expected to be associated with variation in views about aid in Australia and which—as a result—ought to be tested for in my own regression analysis. Following standard convention in the literature, I have grouped these variables together in three types: socio‐demographic traits, information and knowledge, and beliefs.

### 2.1 Sociodemographic Traits

The diminishing marginal utility of income provides cause to anticipate that wealthier people will be more supportive of aid (Milner & Tingley 2010). In line with this, most existing studies have found income (or a related measure) to be correlated with support for aid, with the more affluent being more supportive (Chong & Gradstein 2008; Diven & Constantelos 2009; Paxton & Knack 2012). A number of studies have found a similar positive relationship between education and support for aid (Cheng & Smyth 2016; Chong & Gradstein 2008; Diven & Constantelos 2009; Stern 1998). Existing research has also found some evidence that aid is more popular among younger people (Chong and Gradstein 2008; Paxton and Knack 2012). This may reflect generational changes or simply a lower sense of economic vulnerability associated with youth. Paxton and Knack (2012) contend psychological research showing women to be more likely to hold other‐regarding preferences, which provides cause to anticipate that women will be more supportive of aid. Their empirical findings confirm this. However, both Chong and Gradstein (2008) and Henson and Lindstrom (2013) found gender did not affect views about aid.

The generalised concern with the welfare of others associated with some religious beliefs provides some reason to anticipate that religious people will be more supportive of aid (Paxton and Knack 2012). However, Henson and Lindstrom (2013) found no association between religion and opposition to cutting aid in the United Kingdom. Paxton and Knack (2012) also found no relationship between religiosity and support for aid, although they did find a positive relationship between frequent attendance of religious service and support for aid.

### 2.2 Information and Knowledge

Compared with socio‐demographic traits, fewer studies have included variables associated with information sources and knowledge of current affairs. However, Paxton and Knack (2012) found that time spent watching television (a proxy for news consumption and awareness of international events) was positively associated with support for aid. Similarly, the findings of Diven and Constantelos (2009) suggest that people who are more knowledgeable about international affairs are more supportive of aid. And Paxton and Knack found some evidence to suggest being born overseas (once again a proxy for interest in international affairs) increased support for aid (Paxton & Knack 2012).

### 2.3 Beliefs

Most studies on opinions about aid that have included ideology as an independent variable have found a relationship between left‐leaning political views and greater support for aid (Cheng & Smyth 2016; Chong & Gradstein 2008; Milner & Tingley 2010; Milner & Tingley 2013; Paxton & Knack 2012). This relationship is thought to be a product of general preferences for redistribution on the left, although, interestingly, Henson and Lindstrom (2013) found that concern with domestic poverty was associated with a desire to see aid reduced in the United Kingdom. Other beliefs that have been shown to be positively associated with support for aid include positive attitudes to immigrants and foreigners (Clarke et al. 2014; Minato 2015; Prather 2011), as well as cosmopolitan views and support for multilateralism (Paxton and Knack 2012; Diven and Constantelos 2009).

## 3 Data and Methods

The data used in this paper come from two different public opinion surveys. The first is the ANU Poll, which was conducted by the Social Research Centre in May 2014 on behalf of the Australian National University. The poll was a phone poll conducted by using random digit dialling of both landlines and mobile phones. The in‐sample population for the survey was Australian residents aged 18 and over (McAllister 2014). The sample size was 1,204, although because some responses were missing for some variables, the sample I worked with was smaller. 2In robustness tests reported on in the Appendix S1 report on regressions run on datasets where multiple imputation was used to address the issue of missing responses.


The May 2014 ANU Poll focused on foreign policy and included two questions on attitudes to aid. One of these questions was a very general question about support for aid. Its wording was drawn directly from a question used in surveys in other countries:

Do you generally approve or disapprove of the Australian Government providing aid to poorer countries around the world? (Possible responses were ordinal, from strongly disapprove to strongly approve.)

The second question was about the purpose respondents wanted Australian aid given for:

Do you think Australian government aid should be given primarily on humanitarian grounds, or do you think Australia's commercial and political interests should play a significant part? (Here possible responses were: strongly favour humanitarian; favour humanitarian; favour commercial and political; and strongly favour commercial and political.)

The second dataset came from an omnibus survey conducted by Essential Media. The survey was conducted with a randomly selected sample drawn from an online panel of over 100,000 people, and the final sample size was 1,045. The survey was conducted in March 2015. The question involved differed from the first ANU Poll question in that, rather than asking generally about support for aid, it asked about a specific set of cuts to the aid budget. (Those which occurred in 2015.) It also provided information on the size of the aid budget and was designed explicitly to get respondents thinking about policy trade‐offs. The question asked was as follows:

Every year about 1.2% of Australian federal government spending is spent on foreign aid to poor countries. Recently the government announced that it plans to cut foreign aid by nearly 20% starting in July. They have justified this as a means of preventing government debt rising. Which of the following options would you prefer (each involves equivalent amounts of money)?

1. That aid not be cut and government debt levels increase by a small additional amount next year (approximately 0.4%) as a result of this.
2. That aid not be cut and taxes be raised by a small amount (approximately 0.3%) to produce the same reduction in government debt sought from the aid cut.
3. That aid not be cut and other government expenditure be cut by a small amount (approximately 0.2%) to produce the same reduction in government debt sought from the aid cut.
4. That aid be cut by 20% as the government is currently planning.

Both datasets were weighted to be representative of the Australian voting age population. Both datasets also included a suite of socio‐demographic and belief‐based questions which provided me a set of independent variables that I used in the regression analysis described below. Variables were chosen on the basis of the literature described above. The ANU Poll provided more variables than were available in the Essential Media Dataset. However, a key group of variables were common to both datasets. Table S1 provides descriptive statistics for these variables.

My analysis below starts with simple comparisons of the aggregate responses to the questions listed above. However, in addition to comparing overall results, I used different forms of multiple regression to examine the traits associated with different responses to the three questions. Because the ANU Poll question about approval of Australia giving foreign aid solicited ordinal responses, I used ordered logistic regressions. 3Logistic regressions were used rather than probit models because they can produce results in the form of (somewhat more easily interpretable) odds ratios. In terms of predicted probabilities, the substantive findings that emerge from logistic regressions are nearly identical to those that emerge from probit models (Long & Freese 2014). Using probit models does not change the findings that follow in any substantive way. In the instance of the ANU Poll question about aid purpose, because the response categories were in part categorical (humanitarian versus Australia's interest) and in part ordinal (strongly favour vs favour for each category), I transformed the variable into two categories (humanitarian and Australia's interest) and disregarded the favour versus strongly favour distinction. Having done this, I analysed this question by using a logistic regression.

For the Essential Media Poll question on aid cuts versus alternatives, while multinomial regression using the different categories would have been possible, in the interests of more easily interpretable results, and because the issue of most interest for this study was simply whether Australians favoured aid cuts or not, I opted to convert the response data into a binary variable distinguishing favouring aid cuts from favouring any of the policy alternatives, and subsequently made use of logistic regressions to analyse the data. All the regression models were run with survey weighting applied. Alternative models, run as robustness tests, are reported on in the Appendix S1. Note for now, however, that except when discussed none of the main relationships identified below changed significantly in these robustness tests.

## 4 Results

In this section, I report first on aggregate level responses to all three questions and the differences between them. Then, I report on regression analysis of responses to the ANU Poll question on support for aid. Following this, I report on regression analysis of the responses to the Essential Media question on aid cuts. Finally, I report on the ANU Poll question about the purpose of aid. When reporting regression results, I discuss independent variables in three different groups: those associated with socio‐demographic traits, those associated with information and knowledge, and those associated with beliefs.

### 4.1 Aggregate Opinions

**Figure 1:**
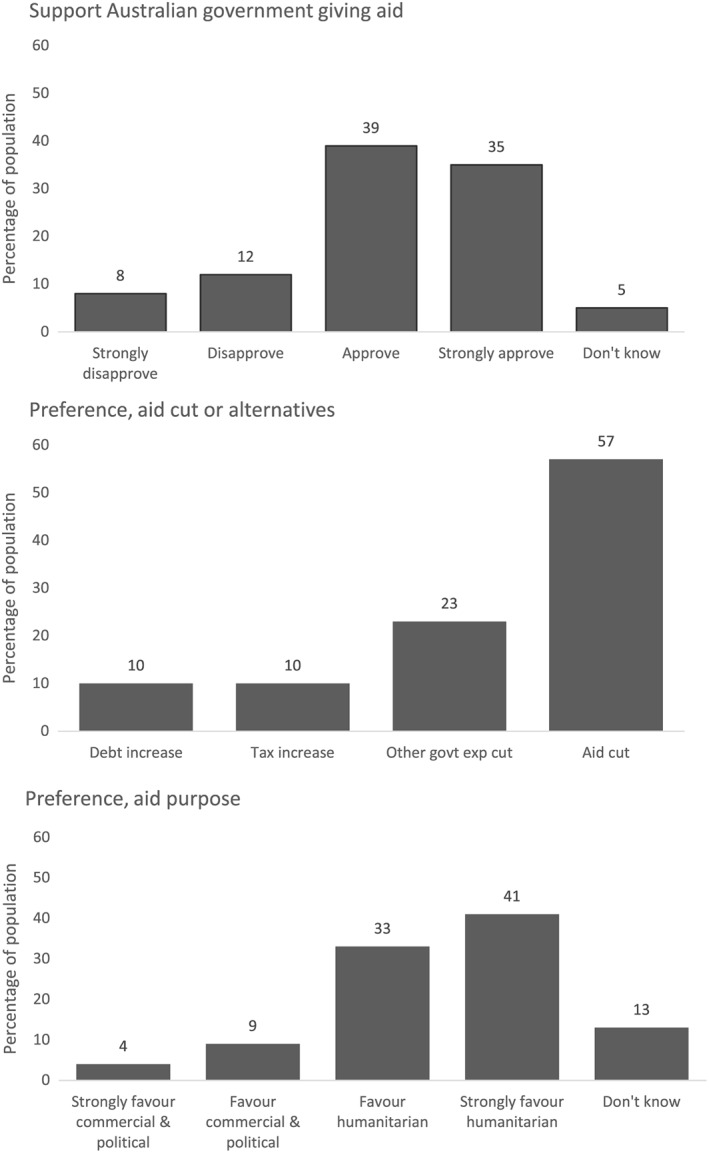
Overall Responses to Questions About Aid

Figure 1 shows the weighted responses to the ANU Poll question on support for the Australian Government giving aid. Figure 1 also shows the weighted responses to the Essential Media question about the 2015 aid cuts. In addition, Figure 1 shows the weighted responses to the ANU Poll question about the purpose of Australian aid. The charts in the figure show that a clear majority of Australians approve of their country giving aid. However, the figure also shows that when Australians were presented with a more specific set of details, and trade‐offs between an aid cut and other policy alternatives, a majority favoured aid cuts. The final chart in the figure shows that a substantial majority of Australians want their country's aid given for humanitarian ends, rather than to benefit Australia.

It is possible, given the ANU Poll and Essential Media surveys were separate surveys, that the differences between responses to the general question about approval of Australia giving aid and the more specific question about aid cuts are simply a product of different samples; however, the magnitude of the differences makes this very unlikely. In the ANU Poll question, 74 per cent of respondents either approve or strongly approve of Australia giving aid, while in the Essential Media question, only 43 per cent of the population opposed aid cuts. 4Reflecting the magnitude of the difference, the p‐value for the difference in a two sample proportion test is <0.01 Although most Australians approve of Australia giving aid in a broad sense, for a sizable subset, this approval did not mean that they were averse to seeing the aid budget reduced. On the other hand, in aggregate, there appears less difference between support for aid and a preference for giving aid for humanitarian reasons rather than giving it to bring benefits to Australia. 5Although differences in question wording make direct comparisons difficult, using more recent data, Wood and Burkot (2017, p. 5) find a public slightly less in favour of aid cuts. Preliminary study of the same data reveals very similar correlates of views on aid volume to those found here.


### 4.2 Predictors of Support for Aid

Moving from aggregate results, Table 1 shows the results of a series of ordered logistic regression models in which the dependent variable comes from the basic ANU Poll question on approval of Australia giving foreign aid. In all regressions, people who responded ‘don't know’ were treated as missing. The coefficients provided are odds ratios; beneath them, *p*‐values are provided in parentheses. 6
*p*‐values are provided rather than standard errors because the usual rule of thumb that allows for inferring *p*‐values from coefficients and standard errors is not applicable when reporting odds ratios. Model 1 includes only socio‐demographic variables. Model 2 adds information‐related variables, and Model 3 includes additional variables to do with beliefs. Model 4 is chosen to emulate, in terms of the independent variables used, the subsequent regressions run on data from the Essential Media question about aid cuts and policy trade‐offs.

**Table 1:** Ordered Logistic Regression Results, Support for Australia Giving Aid

Support Australian Government aid	Model 1	Model 2	Model 3	Model 4
Age	0.99* (0.01)	0.99** (0.01)	0.99* (0.04)	0.99** (0.01)
Academic tertiary education	2.74*** (0.00)	2.70*** (0.00)	1.89** (0.00)	2.70*** (0.00)
Income (natural log)	1.20 (0.06)	1.16 (0.15)	1.25 (0.07)	1.27* (0.02)
Urban	1.53** (0.01)	1.50*(0.02)	1.25 (0.23)	1.42* (0.03)
Male	0.72* (0.02)	0.72* (0.03)	0.62** (0.00)	0.71* (0.02)
Religious never attends (vs non‐religious)	0.52** (0.00)	0.52** (0.00)	0.54** (0.01)	
Religious attends <1/yr	0.46** (0.00)	0.42*** (0.00)	0.51* (0.01)	
Religious attends at least once/year	0.69 (0.16)	0.67 (0.15)	0.66 (0.22)	
Religious attends several times/year	0.74 (0.21)	0.78 (0.31)	0.93 (0.81)	
Religious attends at least once/month	0.43* (0.03)	0.49 (0.07)	0.56 (0.14)	
Religious attends at least once/week	1.70* (0.03)	1.82* (0.03)	2.51** (0.00)	
Born in a developing country		0.76 (0.32)	0.98 (0.94)	
News intake		1.11 (0.51)	1.07 (0.71)	
Worried about immigration			0.70 (0.09)	
Worried about budget			0.62 (0.06)	
Worried about domestic poverty			2.30** (0.00)	
Favourable views of China/Indonesia			1.89***(0.00)	
Favourable views of multilaterals			1.60***(0.00)	
Party Labour (vs Coalition)			2.28*** (0.00)	2.46*** (0.00)
Party Greens (vs Coalition)			2.84** (0.00)	3.17*** (0.00)
Party other (vs Coalition)			0.90 (0.71)	0.93 (0.79)
Party do not know (vs Coalition)			1.53 (0.20)	1.34 (0.32)
Cut 1	0.11*** (0.00)	0.12*** (0.00)	0.11** (0.00)	0.23*** (0.00)
Cut 2	0.33**(0.01)	0.36 (0.07)	0.40 (0.21)	0.71 (0.44)
Cut 3	2.52* (0.03)	2.63 (0.10)	4.62* (0.04)	5.54*** (0.00)
*N*	842	802	762	841

Coefficients are odds ratios.

*** *p* < 0.001.

** *p* < 0.01.

* *p* < 0.05.

Almost all of the socio‐demographic traits included in the regression models are statistically significant and are statistically significant in all of the models. Being older and being male are associated with a lower probability of supporting aid, while having an academic education and living in an urban area are associated with an increased probability of supporting aid, although the urban variable ceases to be statistically significant in the third model.

The relationship between income and support for aid is not statistically significant except in the final model. However, the *p*‐values for this variable are close to 0.05 in three of the other models. 7Moreover, the relationship became statistically significant in three of the four alternate models run as robustness tests and reported on in the Appendix S1. The relationship between religiosity and support for aid is complex: Religious people who rarely attend religious service are less supportive of aid than non‐religious people; however, frequent attenders of religious service are actually more likely to approve of Australia giving aid.

The information‐related variables are not associated with support for aid. However, beliefs are important. The two beliefs that, broadly put, reflect a cosmopolitan or internationalist worldview (favourable views of China and Indonesia, and support for multilateral organisations) are both positively associated with support for aid. Concern with domestic poverty is also positively associated with support for aid. Politically, supporters of the left leaning Green Party and centre‐left Labour party are much more supportive of aid than centre‐right Coalition supporters.

**Figure 2:**
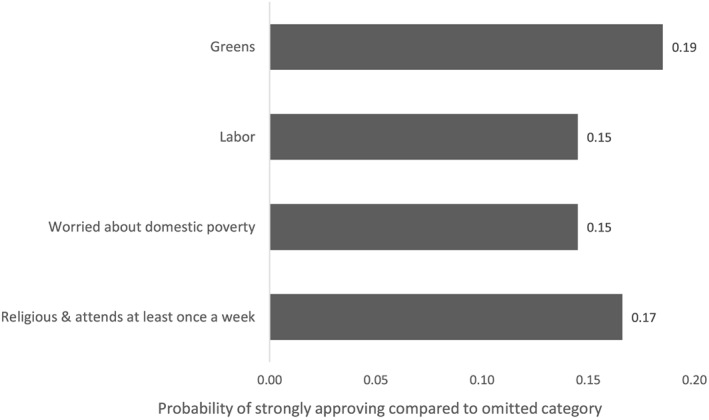
Increase in Probability of Strongly Approving of Australia Giving Aid

The statistically significant predictors in Model 3 all have substantively meaningful effects. In Figure 2, I use predicted marginal effects to illustrate the magnitude of the effect for the largest categorical predictors from Model 3. These are Greens and Labour supporters, people who are worried about domestic poverty and people who attend religious service at least once a week. For each predictor, the chart shows the change in probability of strongly approving of Australia giving aid vis‐à‐vis the omitted category. (The omitted categories are respectively Coalition supporters, people who are not worried about domestic poverty and the non‐religious.) The probabilities in the chart are calculated by using average marginal effects and, to give one example, can be read as showing that, averaged across the population and controlling for other factors, being a Green voter instead of a Coalition voter increases one's probability of strongly approving of Australia giving aid by 0.19.

Three continuous variables in Model 3 of Table 1 are also statistically significant: age, attitudes to multilateral organisations and favourable views about China and Indonesia. Simply glancing at the coefficients for the variables about China and Indonesia, and views about multilaterals, suggests that the effects of these variables are non‐trivial. And, indeed, averaged across the population and controlling for other variables, a one standard deviation change in support for multilaterals is associated with a 0.08 increase in an Australian's probability of strongly approving of aid. The same change in views about China and Indonesia is associated with a 0.11 increase. The size of the coefficient of age, on the other hand, seems to indicate a trivial effect. However, the coefficient is for an additional year of age, and age's impact becomes much more notable over longer periods. On average, with other effects controlled for, the probability of a 60‐year‐old strongly approving of aid is 0.07 lower than that of a 20‐year‐old.

### 4.3 Aid Cuts Versus Alternatives

Having looked at overall support for aid, in this section, I draw on the question asked about the 2015–2016 cuts to the Australian government aid budget. As already discussed and shown in Figure 1, opposition to the 2015–2016 aid cuts was much lower than general support for aid. Table 2 shows the results of two logistic regression models run in which the dependent variable was a binary variable coded zero if the respondent preferred that aid be cut and coded one if the respondent preferred a policy alternative. The coefficients shown are odds ratios. *p*‐values are shown in parentheses. The data provided by Essential Media included fewer potential covariates than was the case with the ANU Poll data. However, a number of important socio‐demographic covariates are included in Model 1 and the belief‐based variable of preferred political party is included in Model 2.

**Table 2:** Logistic Regression Results, Alternative Policy Versus Aid Cuts

	Model 1	Model 2
Age	0.98*** (0.00)	0.98*** (0.00)
Academic tertiary education	1.74*** (0.00)	1.64** (0.00)
Income (natural log)	0.92 (0.51)	0.91 (0.46)
Urban	1.50* (0.02)	1.52* (0.02)
Male	0.83 (0.21)	0.87 (0.36)
Party Labour (vs Coalition)		2.16*** (0.00)
Party Greens (vs Coalition)		4.41*** (0.00)
Party other (vs Coalition)		1.30 (0.37)
Party do not know (vs Coalition)		1.23 (0.41)
Constant	1.92 (0.26)	1.20 (0.77)
N	885	847

Coefficients are odds ratios.

*** *p* < 0.001.

** *p* < 0.01.

* *p* < 0.05.

For the socio‐demographic traits, the results of these regressions are similar but not identical to those from the ANU Poll data on general support for aid (those shown in Model 4 in Table 1). Because the data come from two separate surveys, it is not possible to formally test the differences between coefficients. However, an informal comparison suggests that results for age, academic education, urban dwelling and political views are similar. A similar comparison suggests that the relationship between gender and views about cuts differs from the relationship between gender and general approval of aid giving. It appears that the effect of income differs too.

**Figure 3:**
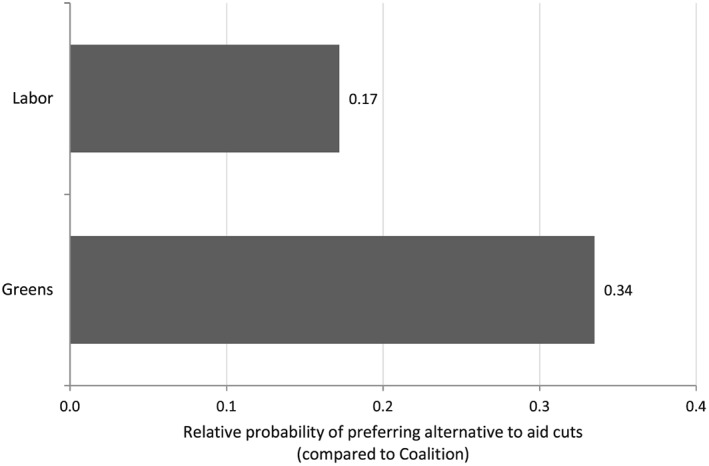
Party and Increase in Probability of Support for Policy Alternatives to Aid Cuts

Figure 3 gives a sense of the substantive magnitude of the effect of party support on opposition to aid cuts. As with Figure 2, the figure is based on calculations using average marginal effects, and it can be interpreted the same way, with the omitted base category being Coalition supporters. As can be seen, as with Figure 2, the impact of party allegiance is substantial. In the case of Green party supporters, it is particularly high.

### 4.4 The Purpose of Aid

Table 3 shows that the results of three logistic regression models run with a dependent variable that was binary and coded one if the respondent favoured or strongly favoured the purpose of Australian aid to be humanitarian, or zero if the respondent favoured or strongly favoured the purpose of Australian aid being to benefit Australia commercially or politically. The first model is limited to socio‐demographic variables, the second includes information‐related variables, and the third includes beliefs. The coefficients shown are odds ratios and *p*‐values are shown in parentheses.

**Table 3:** Logistic Regression Results, Preferences for Aid Purpose

	Model 1	Model 2	Model 3
Age	1.01 (0.09)	1.01 (0.17)	1.02* (0.05)
Academic education	1.12 (0.65)	1.14 (0.60)	0.73 (0.26)
Income (natural log)	1.03 (0.85)	0.93 (0.64)	1.04 (0.82)
Urban	0.74 (0.25)	0.81 (0.43)	0.70 (0.20)
Male	0.54** (0.01)	0.58* (0.03)	0.64 (0.10)
Religious but never attends (vs non‐religious)	0.87 (0.67)	1.04 (0.91)	1.05 (0.89)
Religious and attends <1/yr	0.94 (0.91)	1.01 (0.99)	1.62 (0.46)
Religious and attends at least once a year	1.18 (0.72)	0.92 (0.87)	0.79 (0.63)
Religious and attends several times per year	1.21 (0.62)	1.18 (0.70)	1.32 (0.54)
Religious and attends at least once a month	0.43* (0.04)	0.43 (0.06)	0.55 (0.26)
Religious and attends at least once a week	1.18 (0.67)	1.16 (0.74)	1.72 (0.27)
Born in a developing country		0.52 (0.09)	0.52 (0.13)
News intake		1.88* (0.01)	2.13** (0.01)
Worried about immigration			0.65 (0.16)
Worried about budget			0.78 (0.53)
Worried about domestic poverty‐related issues			6.90** (0.01)
Favourable views of China/Indonesia			1.12 (0.44)
Favourable views of multilaterals			1.47** (0.00)
Party Labour (vs Coalition)			1.98* (0.03)
Party Greens (vs Coalition)			4.40** (0.01)
Party other (vs Coalition)			1.18 (0.69)
Party do not know (vs Coalition)			4.73* (0.02)
Constant	5.15* (0.01)	1.71 (0.54)	0.47 (0.46)
*N*	793	754	720

Coefficients are odds ratios; *p*‐values reported in parentheses.

*** *p* < 0.001.

** *p* < 0.01.

* *p* < 0.05.

Scanning the results table quickly reveals that there are almost no socio‐demographic variables that are related to views about the ends aid should be given for. Of these variables, the apparent relationship in Model 1 between monthly religious service and a preference for aid given to advance Australia's interests is very hard to find an explanation for, given the attendance categories above and below have odds ratios greater than one. Because of this, it seems likely the observed relationship is a product of chance alone. The relationship between being male and a preference for aid given in Australia's interest appears more plausible, although the relationship ceases to be statistically significant in the third model. The relationship between age and support for humanitarian aid that emerges in Model 3 indicates that older Australians are more supportive of humanitarian aid. This is a surprising contrast with the results from the two earlier regressions on support for aid and views about aid cuts, in which older people were less supportive of aid and less likely to oppose aid cuts.

**Figure 4:**
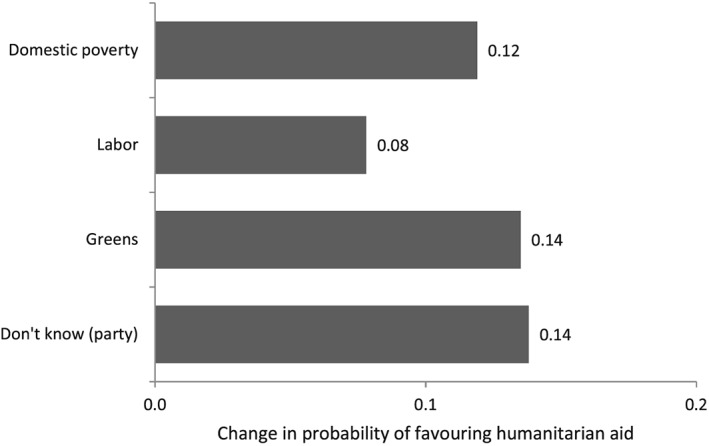
The Impact of Concern With Domestic Poverty and Parties on Aid Purpose

Moving beyond demographic and socioeconomic traits, other findings emerge. The first of these is that people who consume more news are more likely to prefer that Australian aid be given for humanitarian purposes. Of the belief‐related variables, favourable opinions about multilateral institutions are clearly associated with support for aid given for humanitarian ends, as is concern about domestic poverty. Labour supporters and Green supporters are more likely than Coalition supporters (the omitted category) to prefer that aid be given on humanitarian grounds. 8The Labour relationship ceases to be statistically significant in one of the alternate models run as robustness tests. As with earlier regressions, in Figure 4, I illustrate the magnitude of the statistically significant categorical predictors. The *x*‐axis plots the average change in probability of favouring humanitarian aid compared with the omitted category with other variables controlled for.

Because the question about aid purpose was asked in the same survey as the question about approval of aid giving, it is possible to formally test for differences in the coefficients of the independent variables across the full models run for both questions. Such tests show that the relationship between a number of traits and the responses to the two questions are clearly different. Full results of these tests are provided in a table in the online Appendix S1; however, for now, of particular interest is the fact that, as noted above, the relationship between age and the belief that aid should be given for humanitarian ends is positive, whereas the relationship between age and support for aid is negative. Also, academic education is positively associated with support for age but there is no evidence of any relationship between academic education and views about aid purpose. (In formal testing in the Appendix S1, the difference in coefficients for both of these variables is found to be statistically significant.) The subset of the Australian population most likely to approve of Australia giving aid is not identical to that most likely to want Australia to give aid for the sake of helping other countries.

## 5 Discussion

Most Australians approve of their government giving aid; however, responses to a more detailed policy‐related question show that, when confronted with trade‐offs, despite their broad approval of aid, in 2015, the majority of Australians were happy to see the aid budget cut. When faced with a choice between aid cuts and domestic alternatives to aid cuts, most Australians indicated that they preferred the course of action in which aid was cut and costs were borne overseas. At the same time, however, few Australians want a domestic dividend from the aid that their country does give: Most were happy to see this money given for humanitarian ends, rather than for the sake of bringing benefits to Australia. Taken together, these findings have important ramifications for those who would like to see recent changes to Australian aid reversed.

The first of these is that promoting aid by emphasising the benefits that it can bring to Australia is not likely to be an effective strategy for shifting the views the typical Australian holds about the size of the aid budget. Recent research has shown that it is possible to change people's views on aid. However, doing so is not straightforward, and not all arguments succeed (Wood 2016). At present, the benefits that aid can bring to Australia are being repeatedly emphasised by supporters of aid within the Coalition government. Senior supporters of aid within the Coalition government have explicitly stated that they believe that doing this is needed to win support for an increased aid budget in the future (Bishop 2017; Fierravanti‐Wells 2017). My findings do not do not provide any evidence that Australians are particularly likely to be amenable to arguments made along these lines.

The second ramification is that it will likely be easier to enlist the public to the cause of change in the area of aid purpose than it will be to the cause of an increased an aid budget. To date, however, public campaigning on aid‐related matters, which has been led by the NGO coalition the Campaign for Australian Aid, has focused almost exclusively on attempts to reverse aid cuts (e.g. Campaign for Australian Aid 2017). There are compelling arguments for increasing the Australian aid budget, and policy is rarely changed without pressure. As a result, advocacy about aid volume has its place. However, given that the public is more likely to be supportive of changes to the purpose of Australian aid aimed at increasing its development focus, aid's advocates would be wise to devote some of their public campaigning resources to this area.

The differences in the findings between the regressions on support for aid and views about aid purpose suggest that—if it is undertaken—campaigning on purpose may be better targeted at a different subsection of the Australian population than that targeted in campaigning on aid volume. To date, much of the work of organisations like the Campaign for Australian Aid has been focused on university campuses. My findings suggest this is appropriate given the Campaign's focus on aid volume, and given its desire to motivate an already supportive base. However, were campaigners to shift their focus to the purpose of aid, my results indicate that the most supportive base for this particular issue may be found elsewhere. Of course, as Figure 1 shows, support on the issue of aid purpose is high across Australia, and university students may bring other advantages, including a higher propensity towards taking action. However, at the very least, other demographics ought to be easier to motivate if campaign focus is shifted to aid purpose. As a result, increased interaction with other parts of Australian society should be considered if the focus of campaigning does shift to aid purpose.

The clear left–right divide that was found in all three sets of regression results provides campaigners with a clear steer as to where their ideological base is most likely to be found. Knowledge of a support base among people on the centre–left may seem of limited utility at a time when a centre–right coalition currently holds government. However, governments change, and those who wish to promote high‐level policy change would do well to prepare to mobilise public support on both aid quality and aid quantity issues from the left when the government next changes. Such a political break could provide the best opportunity to reverse recent changes and ensuring that there is a loud public push for change from among the central support base of the new government will be important.

Public opinion is not the only factor influencing high‐level Australian aid policy, but international evidence suggests it can play a role. There is also a clear belief both among campaigners and Australian politicians that the views of the public matter. This paper has shown what these views are, while at the same time showing that the Australian public is far from homogenous in its views. Different types of Australians have different views on policy‐related questions about aid, and individual Australians do not always hold the same views about all aspects of aid policy.

## Supporting information

Appendix Table S1 Details and descriptive statistics, ANU Poll
Appendix Table S2 Details and descriptive statistics, Essential Media
Appendix Table S3 Robustness tests, general support for aid
Appendix Table S4 Robustness tests, aid cuts
Appendix Table S5 Robustness tests, aid purpose
Appendix Table S6 Differences in coefficients for aid approval and aid purpose
