# Supplementary material for: Aid Policy and Australian Public Opinion
Source: Asia Pac Policy Stud. 2018 Apr 16;5(2):235–48. doi: 10.1002/app5.230 (PMC6049982; doi:10.1002/app5.230)
Supplement: Supplementary file 1 — Appendix Table S1 Details and descriptive statistics, ANU Poll Appendix Table S2 Details and descriptive statistics, Essential Media Appendix Table S3 Robustness tests, general support for aid Appendix Table S4 Robustness tests, aid cuts Appendix Table S5 Robustness tests, aid purpose Appendix Table S6 Differences in coefficients for aid approval and aid purpose [file APP5-5-235-s001.docx]

# Appendix 1

The following two tables detail the variables from the ANU Poll and Essential Media surveys that were used as independent variables in the regression analyses in this paper.

**Appendix Table 1 Details and descriptive statistics, ANU Poll**

| **Variable** | **Detail** |  |  |  |  |
| --- | --- | --- | --- | --- | --- |
| ***Sociodemographic*** |  | **Mean** | **Std Dvn** | **Min** | **Max** |
| Age | Age in years. | 47.2 | 17.9 | 18 | 93 |
| Income | Income: thousands per adult in the household (natural log). | 80.3 | 54.5 | 10 | 175 |
| Urban | A dummy variable coded one if the respondent lived in an urban area. | 0.7 | 0.5 | 0 | 1 |
| Male | Gender (with male coded as one). | 0.5 | 0.5 | 0 | 1 |
|  |  |  |  |  |  |
| Academic Education | A dummy variable coded one if the respondent had an academic tertiary education. | 0.4 | 0.5 | 0 | 1 |
| Religious | A categorical variable based on religiosity and frequency of attendance if religious. | **Percent** |  |  |  |
| Not religious |  | 36.8 |  |  |  |
| Religious never attends |  | 17.9 |  |  |  |
| Attends < once a year |  | 4.5 |  |  |  |
| At least once a year |  | 8 |  |  |  |
| Several times a year |  | 11.8 |  |  |  |
| At least once a month |  | 5.9 |  |  |  |
| At least once a week |  | 15.1 |  |  |  |
| News Intake | A constructed variable calculated as the average response given by the respondent to how often they consumed a range of news media (newspapers, free to air television, pay television, radio, print newspapers, online newspapers, social media and other online sources). | 2.5 | 0.5 | 1 | 4 |
| Developing Country | A dummy variable coded one if the respondent had been born in a developing country. | 0.1 | 0.3 | 0 | 1 |
| Views about China/Indonesia | A variable, constructed using principal component factor analysis, to capture attitudes regarding two of Australia’s largest developing country neighbours: Indonesia and China. | 0 | 1 | -3 | 2.1 |
| Views of Multilaterals | A variable, constructed using principal component factor analysis, derived from respondents’ answers to questions about the United Nations, the United Nations Development Program, the World Health Organization, the World Bank and the World Trade Organization. | 0 | 1 | -3 | 2.5 |
| Worried about Immigration | A dummy variable which was coded one if, when asked to name the most, or second-most, important issue facing Australia today, the respondent stated ‘immigration’. | 0.1 | 0.4 | 0 | 1 |
| Worried about Budget | A dummy variable which was coded one if, when asked to name the most, or second-most, important issue facing Australia today, the respondent stated ‘the budget’. | 0.1 | 0.3 | 0 | 1 |
| Domestic Poverty | A dummy variable coded one if the respondent stated that poverty was the most or second-most significant issue facing Australia. | 0.3 | 0.5 | 0 | 1 |
| Party | A categorical variable based on the party the survey respondent said they were most likely to vote for in an election ‘held today’. | **Percent** |  |  |  |
| Coalition |  | 32.7 |  |  |  |
| Labor |  | 32.2 |  |  |  |
| Greens |  | 13.2 |  |  |  |
| Other |  | 13 |  |  |  |
| Don't Know |  | 8.9 |  |  |  |

**Appendix Table Table 2 Details and descriptive statistics, Essential Media**

| **Variable** | **Detail** |  |  |  |  |
| --- | --- | --- | --- | --- | --- |
| ***Sociodemographic*** |  | **Mean** | **Std Dvn** | **Min** | **Max** |
| Age | Age in years. | 44.1 | 15.8 | 19 | 70 |
| Household income | Pre-tax household income (natural log). | 68 | 32.3 | 3.9 | 110 |
| Urban | A dummy variable (an approximation of urban) depending on whether the respondent lived in a state capital or not (with urban coded as one). | 0.6 |  |  |  |
| Male | Gender (with male coded as one). | 0.5 |  |  |  |
| Academic Education | A dummy variable coded one if the respondent had an academic tertiary education. | 0.4 |  |  |  |
| ***Belief*** |  | **Percent** |  |  |  |
| Party | A categorical variable based on party preference. |  |  |  |  |
| Coalition |  | 35.3 |  |  |  |
| Labor |  | 33.7 |  |  |  |
| Greens |  | 7.5 |  |  |  |
| Other |  | 8.8 |  |  |  |
| Don't Know |  | 14.7 |  |  |  |

# Appendix 2: Robustness tests

In this appendix I detail the robustness tests that I ran and their outcomes.

### *General*

Tests for multicollinearity suggested it was not an issue in any of the models used.

### *General support for aid*

Where I ran ordered logistic regression models (specifically, the models used to test associations with general support for aid) I tested to see if the parallel lines assumption was violated by the models. Tests of the first three models did not reveal any violations, and the fourth model did not violate the assumption as whole; however, for one variable, that associated with support for the Green Party, there was a statistically significant violation. As a consequence I re-ran this particular model as a generalised ordered logit regression (Williams 2006). When I did this there were no substantive changes to the coefficients of any variables except that for Green Party support. And even for Green Party support, the key finding – that Green Party support was associated with a higher propensity to support or strongly support Australia giving aid – was unchanged.

Appendix Table 3 shows the outcomes of robustness tests run on the ANU Poll general support for aid data. Variations from the main findings are shaded light grey. As can be seen most of the original findings withstand robustness tests well. All tests were run as variants of the full model from the results in the main text.

**Appendix Table 3 Robustness tests, general support for aid**

|  | Original | Parsimonious | Alternate Dep. Var. | Multiple Imputn. |
| --- | --- | --- | --- | --- |
| Age | 0.99* | 0.99* | 0.99 | 0.99* |
|  | (0.04) | (0.05) | (0.10) | (0.02) |
| Academic tertiary education | 1.89** | 2.01*** | 1.70** | 1.85*** |
|  | (0.00) | (0.00) | (0.01) | (0.00) |
| Income (natural log) | 1.25 | 1.26* | 1.28* | 1.25* |
|  | (0.07) | (0.04) | (0.04) | (0.05) |
| Urban | 1.25 | 1.30 | 1.30 | 1.29 |
|  | (0.23) | (0.14) | (0.14) | (0.12) |
| Male | 0.62** | 0.64** | 0.64** | 0.62** |
|  | (0.00) | (0.01) | (0.01) | (0.00) |
| Religious but never attends | 0.54** | 0.55** | 0.56** | 0.61* |
|  | (0.01) | (0.01) | (0.01) | (0.02) |
| Religious attends < 1/year | 0.51* | 0.59 | 0.56* | 0.51** |
|  | (0.01) | (0.06) | (0.03) | (0.01) |
| Religious attends at least once a year | 0.66 | 0.70 | 0.69 | 0.69 |
|  | (0.22) | (0.26) | (0.24) | (0.23) |
| Religious attends several times per year | 0.93 | 0.88 | 1.00 | 0.96 |
|  | (0.81) | (0.64) | (1.00) | (0.88) |
| Religious attends at least once a month | 0.56 | 0.56 | 0.62 | 0.58 |
|  | (0.14) | (0.11) | (0.21) | (0.14) |
| Religious attends at least once a week | 2.51** | 2.39** | 2.39** | 2.30** |
|  | (0.00) | (0.00) | (0.00) | (0.00) |
| Born in a developing country | 0.98 |  | 0.98 | 1.12 |
|  | (0.94) |  | (0.96) | (0.67) |
| News intake | 1.07 | 1.04 | 1.11 | 1.18 |
|  | (0.71) | (0.82) | (0.57) | (0.31) |
| Worried about immigration | 0.70 | 0.75 | 0.72 | 0.88 |
|  | (0.09) | (0.17) | (0.11) | (0.53) |
| Worried about budget | 0.62 | 0.68 | 0.57* | 0.61* |
|  | (0.06) | (0.11) | (0.03) | (0.04) |
| Worried about domestic poverty | 2.30** | 2.14** | 2.48*** | 1.94** |
|  | (0.00) | (0.00) | (0.00) | (0.01) |
| Favourable views of China/Indonesia | 1.89*** | 1.83*** | 1.85*** | 1.82*** |
|  | (0.00) | (0.00) | (0.00) | (0.00) |
| Favourable views of multilaterals | 1.60*** | 1.52*** | 1.60*** | 1.51*** |
|  | (0.00) | (0.00) | (0.00) | (0.00) |
| Party - Labor (versus Coalition) | 2.28*** | 2.25*** | 2.06*** | 2.23*** |
|  | (0.00) | (0.00) | (0.00) | (0.00) |
| Party - Greens (versus Coalition) | 2.84** | 2.98*** | 2.92*** | 3.03*** |
|  | (0.00) | (0.00) | (0.00) | (0.00) |
| Party - Other (versus Coalition) | 0.90 | 0.97 | 0.92 | 0.92 |
|  | (0.71) | (0.90) | (0.74) | (0.71) |
| Party - Don't Know (versus Coalition) | 1.53 | 1.55 | 1.72 | 0.88 |
|  | (0.20) | (0.19) | (0.09) | (0.64) |
| N | 762 | 799 | 786 | 1010 |

This model is included in the table (‘Original’) as well as the following variants of the this model: a more parsimonious version of the model in which (following Gelman & Hill 2006, p. 69) I excluded all independent variables that were not statistically significant and had a different sign from that expected from theory; a version of the model in which ‘Don’t know’ responses were coded as neutral rather than missing; and the results of regressions run on a dataset generated using multiple imputation to address potential issues associated with missing values for the natural log of income and for answers to the question about support for aid.^[[1]](#footnote-1)^

### *Aid cuts*

A link test run on the full aid cuts model suggested it was well specified as did the Hosmer-Lemeshow goodness of fit statistic. Testing showed no issues with high-leverage outliers.

Appendix Table 4 shows the results of robustness tests run making use of alternate versions of the aid cuts regressions. The first column shows the original (full) model. The second shows a more parsimonious version of the model (as per above). The third version shows the results of regressions run after the multiple imputation of missing values for income and party. Significant differences from the main model are not shaded grey in this instance as they do not exist.

**Appendix Table 4 Robustness tests, aid cuts**

|  | Original Model | Parsimonious model | Multiple Imputation |
| --- | --- | --- | --- |
| Age | 0.98*** | 0.98*** | 0.98*** |
|  | (0.00) | (0.00) | (0.00) |
| Academic Tertiary Education | 1.64** | 1.64** | 1.69*** |
|  | (0.00) | (0.00) | (0.00) |
| Income (natural log) | 0.91 |  | 0.94 |
|  | (0.46) |  | (0.60) |
| Urban | 1.52* | 1.40* | 1.37* |
|  | (0.02) | (0.04) | (0.05) |
| Male | 0.87 | 0.82 | 0.85 |
|  | (0.36) | (0.19) | (0.25) |
| Party - Labor (versus Coalition) | 2.16*** | 2.53*** | 2.58*** |
|  | (0.00) | (0.00) | (0.00) |
| Party - Greens (versus Coalition) | 4.41*** | 4.68*** | 4.69*** |
|  | (0.00) | (0.00) | (0.00) |
| Party - Other (versus Coalition) | 1.30 | 1.61 | 1.61 |
|  | (0.37) | (0.07) | (0.06) |
| Party - Don't Know (versus Coalition) | 1.23 | 1.40 | 1.43 |
|  | (0.41) | (0.14) | (0.11) |
| N | 847 | 994 | 1045 |

### *Aid purpose*

Link tests of the full aid purpose regression model suggested it was reasonably well specified as did the Hosmer–Lemeshow goodness of fit statistic. Appendix Table 5 shows the results of alternate versions of the aid purpose regressions. First it displays the original model. The second column shows the outcomes of a parsimonious regression in which variables were dropped as per the rules outlined above. The third column reports on the results of my full regression model run with high leverage outliers excluded. (Note, however, that examination of outliers suggested no obvious reason why a regression model with them excluded should be preferred to one with them in.) The fourth column reports on the results of regressions run after multiple imputation was used on missing values (as in the aid support regressions). Once again significant changes are shaded in grey.

**Appendix Table 5 Robustness tests, aid purpose**

|  | Original | Parsimonious model | High leverage outliers out | Multiple Imputation |
| --- | --- | --- | --- | --- |
| Age | 1.02* | 1.03** | 1.02* | 1.02* |
|  | (0.05) | (0.00) | (0.04) | (0.02) |
| Academic tertiary education | 0.73 |  | 0.83 | 0.80 |
|  | (0.26) |  | (0.56) | (0.42) |
| Income (natural log) | 1.04 | 0.99 | 0.92 | 0.98 |
|  | (0.82) | (0.92) | (0.64) | (0.90) |
| Urban | 0.70 |  | 0.66 | 0.94 |
|  | (0.20) |  | (0.17) | (0.82) |
| Male | 0.64 | 0.65 | 0.64 | 0.56* |
|  | (0.10) | (0.09) | (0.11) | (0.02) |
| Religious but never attends | 1.05 | 0.96 | 1.09 | 1.18 |
|  | (0.89) | (0.90) | (0.82) | (0.61) |
| Religious and attends < 1/year | 1.62 | 1.40 | 3.31 | 1.97 |
|  | (0.46) | (0.57) | (0.13) | (0.29) |
| Religious and attends at least once a year | 0.79 | 1.04 | 0.89 | 0.69 |
|  | (0.63) | (0.94) | (0.82) | (0.36) |
| Religious and attends several times per year | 1.32 | 1.03 | 1.02 | 1.04 |
|  | (0.54) | (0.94) | (0.96) | (0.92) |
| Religious and attends at least once a month | 0.55 | 0.53 | 0.62 | 0.64 |
|  | (0.26) | (0.18) | (0.34) | (0.35) |
| Religious and attends at least once a week | 1.72 | 1.65 | 3.04* | 2.07 |
|  | (0.27) | (0.25) | (0.03) | (0.10) |
| Born in a developing country | 0.52 |  | 0.31** | 0.65 |
|  | (0.13) |  | (0.01) | (0.31) |
| News intake | 2.13** | 1.73* | 2.49** | 1.90** |
|  | (0.01) | (0.04) | (0.00) | (0.01) |
| Worried about immigration | 0.65 | 0.80 | 0.62 | 0.66 |
|  | (0.16) | (0.45) | (0.16) | (0.13) |
| Worried about budget | 0.78 | 0.89 | 1.00 | 0.96 |
|  | (0.53) | (0.77) | (1.00) | (0.92) |
| Worried about domestic poverty | 6.90** | 7.92** | 9.32* | 3.75* |
|  | (0.01) | (0.00) | (0.03) | (0.02) |
| Favourable views of China/Indonesia | 1.12 | 1.13 | 1.09 | 1.00 |
|  | (0.44) | (0.38) | (0.53) | (0.99) |
| Favourable views of multilaterals | 1.47** | 1.48** | 1.40* | 1.42** |
|  | (0.00) | (0.00) | (0.01) | (0.00) |
| Party - Labor (versus Coalition) | 1.98* | 1.40 | 3.02*** | 1.74* |
|  | (0.03) | (0.26) | (0.00) | (0.05) |
| Party - Greens (versus Coalition) | 4.40** | 4.46** | 15.06*** | 4.26** |
|  | (0.01) | (0.01) | (0.00) | (0.00) |
| Party - Other (versus Coalition) | 1.18 | 1.31 | 2.02 | 1.68 |
|  | (0.69) | (0.50) | (0.07) | (0.17) |
| Party - Don't Know (versus Coalition) | 4.73* | 2.01 | 16.50** | 2.39 |
|  | (0.02) | (0.23) | (0.01) | (0.10) |
| N | 720 | 762 | 706 | 1010 |

# Appendix 3: Formal comparison of aid purpose coefficients with approval of aid coefficients

Because the question about aid purpose was asked in the same survey as the question about approval of aid giving, it is possible to formally test for differences in the coefficients of the independent variables across the full models run both questions. This was done using seemingly unrelated estimates and then F-tests of the difference between individual coefficients. Results are reported on in Appendix Table 6 below.

**Appendix Table 6 Differences in coefficients for aid approval and aid purpose**

| **Variable** | **F** | **p** |
| --- | --- | --- |
| Age | 7.65 | 0.01 |
| Academic | 8.20 | 0.00 |
| Income | 0.82 | 0.36 |
| Urban | 3.31 | 0.07 |
| Gender | 0.02 | 0.89 |
| Religious but never attends | 2.49 | 0.11 |
| Religious - attends < 1/year | 2.68 | 0.10 |
| Religious - attends at least once a year | 0.11 | 0.75 |
| Religious - attends several times per year | 0.46 | 0.50 |
| Religious - attends at least once a month | 0.00 | 0.97 |
| Religious - attends at least once a week | 0.54 | 0.46 |
| Born in a developing country | 1.72 | 0.19 |
| News Intake | 4.43 | 0.04 |
| Worried about immigration | 0.04 | 0.85 |
| Worried about budget | 0.22 | 0.64 |
| Worried about domestic poverty | 1.96 | 0.16 |
| Favourable views of multilaterals | 0.34 | 0.56 |
| Favourable views of China/Indonesia | 9.52 | 0.00 |
| Labor | 0.15 | 0.70 |
| Greens | 0.51 | 0.48 |
| Other | 0.33 | 0.56 |
| Don't know | 2.33 | 0.13 |

# References

Gelman, A and Hill, J (2006) *Data analysis using regression and multilevel/hierarchical models*. Cambridge University Press, Cambridge.

Williams, R (2006) Generalized Ordered Logit/Partial Proportional Odds Models for Ordinal Dependent Variables. *The Stata Journal* 6(1), 58-82.

1. The form of imputation I used was multivariate imputation using chained equations, and the values of the missing variables were imputed using all of the other variables used in the full regression model. [↑](#footnote-ref-1)
